# Manifold lateralisation and variability in the language connectome at 7T

**DOI:** 10.21203/rs.3.rs-9267400/v1

**Published:** 2026-05-05

**Authors:** Lilit Dulyan, Cesare Bortolami, Eva Guzmán Chacón, Ahmad Beyh, Michel Thiebaut de Schotten, Stephanie J Forkel

**Affiliations:** 1 Donders Institute for Brain Cognition Behaviour, Radboud University, Nijmegen, The Netherlands; 2 Max Planck institute for Psycholinguistics, Nijmegen, The Netherlands; 3 Brain Connectivity and Behaviour Laboratory, France; 4 Optical Approaches to Brain Function Laboratory, Istituto Italiano di Tecnologia, Genova, Italy; 5 University of Genova, Genova, Italy; 6 Department of Psychiatry, Brain Health Institute, Rutgers University, Piscataway, NJ, USA; 7 Groupe d’Imagerie Neurofonctionnelle, IMN, CNRS, University of Bordeaux, France

**Keywords:** structural asymmetry, language, interindividual variability, high-field neuroimaging, tractography, connections, white matter, variability index, VI

## Abstract

Language lateralisation has long been viewed as a hallmark of left hemisphere specialisation. Yet the structural foundations of this asymmetry remain unclear. Using ultra-high-field 7T diffusion MRI from 172 Human Connectome Project participants, we reconstructed seven language-recruited white matter tracts and characterised the architecture of hemispheric asymmetry by combining a new Variability Index, Bayesian inference, and manifold learning. Joint analysis across tracts reveals that individuals vary along a continuous manifold of structural lateralisation rather than forming discrete left, right, or bilateral lateralisation phenotypes. Additionally, lateralisation is robust but tract-specific: the arcuate long segment, inferior longitudinal fasciculus, and frontal aslant tract show leftward asymmetry, while the anterior arcuate segment and uncinate fasciculus show rightward asymmetry. Despite these population-level results, microstructural variability is substantial, with certain tracts exhibiting greater dispersion in one hemisphere than the other. Bayesian evidence indicated that structural lateralisation is unrelated to handedness or language performance. Together, these results redefine hemispheric specialisation as a graded rather than distinctly distributed language connectome, balancing conserved structural scaffolds with individual flexibility.

## Introduction

Functional language lateralisation is one of the most distinctive organisational features of the human brain^1–3^. Yet its structural basis remains incompletely understood. While classical models emphasised left-hemispheric dominance^4–6^, diffusion-weighted imaging tractography has revealed that the tracts supporting language, such as the arcuate fasciculus, inferior fronto-occipital fasciculus, uncinate fasciculus, inferior longitudinal fasciculus, and frontal aslant tract, exhibit varying degrees of asymmetry across individuals^7–11^. This variability has prompted speculation that lateralisation occurs not only in strength (a numerical variable) but also in type (a categorical variable), with some individuals showing left- or right-dominant profiles and others more symmetrical patterns. However, whether these individual differences reflect discrete subpopulations or a continuum of variation has never been empirically tested at the level of white matter architecture.

To characterise tract-specific lateralisations and variability with maximal anatomical precision, it is now feasible to leverage high-resolution diffusion MRI datasets and advanced tractography approaches that go well beyond standard tensor models^14^. By reconstructing major language pathways using spherical deconvolution tractography^15–17^ on the multishell, 1 millimetre isotropic data from the Human Connectome Project^18^, it is now possible to obtain improved estimates of fibre orientation and microstructural integrity within each hemisphere. This level of detail enables quantification of subtle lateralisation patterns in association tracts that are often blurred, underestimated, or forgotten in conventional diffusion tractography frameworks^19^. Recent methodological advances have demonstrated that high-angular resolution and fixel-based analyses can capture fine-grained tract profiles and individual variability^20,21^. However, despite these methodological advances, a comprehensive investigation of the lateralisation of language pathways has yet to be achieved.

Here, we combined advanced white matter anatomical dissections^22^ with Bayesian modelling^23^, non-parametric permutation testing^24^, and manifold learning via Uniform Manifold Approximation and Projection (UMAP)^25^ to quantify and visualise the full landscape of white matter lateralisation across language pathways. Bayesian statistics were chosen to enable inference about both the presence and absence of asymmetries^26^, while non-parametric permutation testing^27–29^ allows robust assessment of variability without assumptions about underlying data distribution, and UMAP provides a data-driven, low-dimensional view of the population structure^30^. Together, these complementary approaches allow us to test central tendencies in lateralisation, variability of white matter pathways, and whether lateralisation patterns cluster into distinct subgroups or instead vary continuously across individuals. Through the exploration of the nature of interindividual variability in the language connectome^31^, we aim to clarify a fundamental property of human brain organisation and its implications for interindividual variability in cognition.

## Results

**Figure 1.**
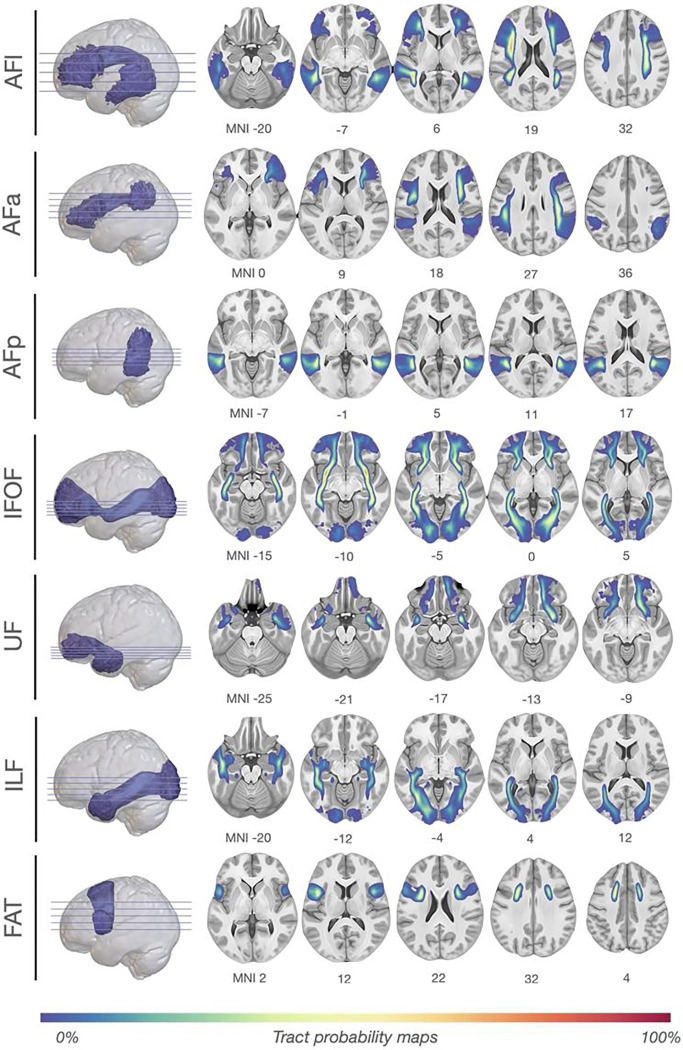
Tract probability maps for seven language white matter pathways. Each panel displays axial slices in MNI space showing the spatial distribution and overlap of binarised tract visitation maps for seven association pathways reconstructed bilaterally: the anterior (AFa), long (AFl), and posterior (AFp) segments of the arcuate fasciculus; the inferior fronto-occipital fasciculus (IFOF); the inferior longitudinal fasciculus (ILF); the uncinate fasciculus (UF); and the frontal aslant tract (FAT). Voxel intensities reflect the proportion of participants (n = 172) in whom a given voxel was traversed by streamlines assigned to each tract, smoothed with a 1 mm FWHM Gaussian kernel to enhance spatial coherence. The colour scale ranges from low (blue) to high (red) group consistency. These group-average maps offer a visualisation of the anatomical consistency and spatial variability of tract reconstructions across individuals.

**Figure 2.**
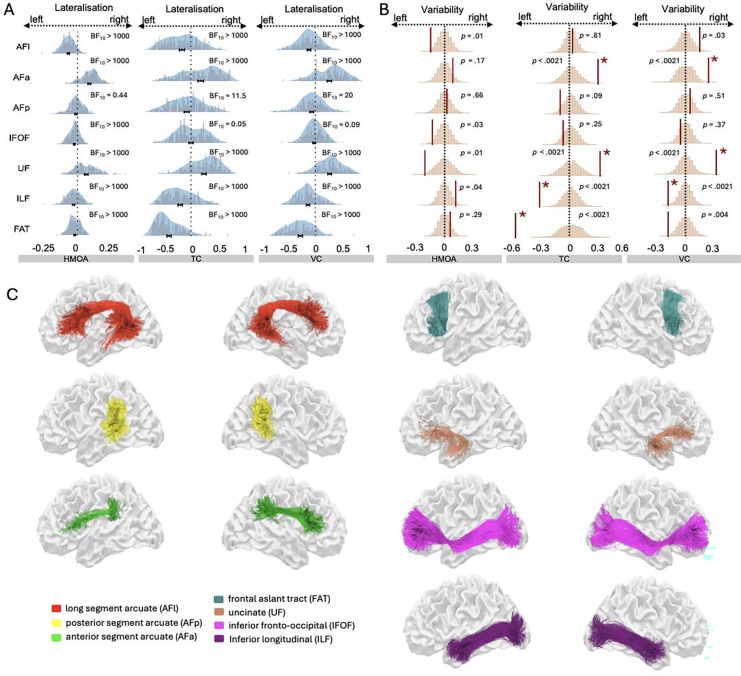
Hemispheric asymmetry in lateralisation and variability in language-recruited tracts across macrostructural and microstructural properties. **A:** Lateralisation in language-recruited tracts. Grey histograms and overlaid blue density plots illustrate the distribution of LI values across participants (n=164). Negative LI values indicate leftward lateralisation; positive values indicate rightward lateralisation. **B**: Asymmetry in the within-hemisphere variability of language-recruited tracts. The X-axis shows the distribution of the permuted Variability Index (VI) values under the null hypothesis of equal variability between hemispheres, based on 100,000 permutations. The Y-axis reflects the frequency of each permuted VI value. Blue curves represent the null distribution; red solid lines mark the observed VI. Negative VI values indicate greater variability in the left hemisphere; positive values indicate greater variability in the right. A value of zero denotes equal variability. The further the observed VI deviates from zero, the less likely it is to have occurred by chance. Red asterisks mark statistically significant results after Bonferroni correction (p < 0.05/24). **C:** 3D reconstruction of seven bilateral white matter pathways. For visualisation, tractograms from 13 randomly selected participants were combined (see Radboud Data Repository for tractograms from all 164 participants, https://doi.org/10.34973/e0fm-pr30). HMOA: Hindrance modulated orientational anisotropy; TC: track count; VC: voxel count; BF_10_ = Bayes factor.

Whole-brain deterministic spherical deconvolution tractograms were reconstructed from 7T diffusion-weighted images of 172 healthy young adult participants from the Human Connectome Project^32^ (https://www.humanconnectome.org/). Seven association tracts consistently recruited in language processing were manually delineated in both hemispheres: the long (AFl), anterior (AFa), and posterior (AFp) segments of the arcuate fasciculus, the inferior fronto-occipital fasciculus (IFOF), uncinate fasciculus (UF), inferior longitudinal fasciculus (ILF), and the frontal aslant tract (FAT, see Figure 1 and Figure 2C). For each tract and hemisphere, macrostructural (voxel count, VC; track count, TC) and microstructural (hindrance modulated orientational anisotropy, HMOA) metrics were extracted. Descriptive statistics and normative ranges of all diffusion indices and the percentage of successful reconstructions per tract are provided in Supplementary Tables 1–2 and Supplementary Figures 1–2.

### Population-level lateralisation of language-recruited tracts

Bayesian modelling was applied to lateralisation indices (LI), quantified as $LI=\frac{{\mathrm{value}}_{\mathrm{right}}\;-\;{\mathrm{value}}_{\mathrm{left}}}{{\mathrm{value}}_{\mathrm{right}}\;+\;{\mathrm{value}}_{\mathrm{left}}}$ (see Methods), to statistically test population-level central tendencies in hemispheric lateralisation. The analyses demonstrated robust and tract-specific hemispheric asymmetries across all language-recruited white matter pathways (Table 1, Figure 2A). Extreme evidence supported leftward lateralisation of the long segment of the arcuate fasciculus (AFl), the inferior longitudinal fasciculus (ILF), and the frontal aslant tract (FAT) across all structural metrics (Bayes factors > 10^4^). In contrast, the uncinate fasciculus (UF) and the anterior segment of the arcuate fasciculus (AFa) showed extreme rightward asymmetry, with Bayes factors exceeding 10^7^ for hindrance-modulated orientational anisotropy (HMOA), track count, and voxel count (Table 1 and Figure 2A).

More variable patterns emerged for the inferior fronto-occipital fasciculus (IFOF) and the posterior arcuate fasciculus (AFp). For the IFOF, leftward asymmetry was supported by extreme evidence for HMOA (BF_10_ = 3.6 × 10^10^), while strong evidence for H_0_ was observed for voxel count and tract count (BF_10_ = 0.05 and 0.09, respectively), indicating an absence of asymmetry and suggesting bilateral organisation. The AFp showed anecdotal evidence for H_0_ for HMOA (BF_10_ = 0.44), whereas track and voxel counts provided strong evidence for leftward asymmetry (BF_10_ = 11.5 and 20.2, respectively) but with 95% credible intervals approaching zero (see Table1).

Together, these results reveal a tract-specific lateralisation architecture: some pathways (AFl, ILF, FAT) show consistent leftward population-level asymmetry, others (AFa, UF) invert that pattern, and a subset (AFp, IFOF) approximate bilateral configuration. This diversity underscores that hemispheric specialisation in the language connectome is not uniform but organised along a continuum of lateralisation strengths and directions.

### Interhemispheric variability

The lateralisation index (LI), computed per individual as $LI=\frac{{\mathrm{value}}_{\mathrm{right}}\;-\;{\mathrm{value}}_{\mathrm{left}}}{{\mathrm{value}}_{\mathrm{right}}\;+\;{\mathrm{value}}_{\mathrm{left}}}$, captures asymmetry in the magnitude of a tract metric: it reflects how much larger or smaller that measure is in one hemisphere relative to the other. Because LI is an individual-level measure, its distribution across participants can itself be characterised by central tendency and spread, offering some window into population-level variability in lateralisation. However, LI does not speak to a related but distinct question: whether the two hemispheres differ in how variable the underlying metric is across individuals. To assess this, we introduced a novel Variability Index (VI), defined as $VI=\frac{{\mathrm{MAD}}_{\mathrm{right}}\;-\;{\mathrm{MAD}}_{\mathrm{left}}}{{\mathrm{MAD}}_{\mathrm{right}}\;+\;{\mathrm{MAD}}_{\mathrm{left}}}$, where MAD is the median absolute deviation from the sample median, computed separately for each hemisphere. While LI asks how different the left and right hemispheres are on a given measure, VI asks whether one hemisphere is more consistent than the other across individuals, i.e., it captures asymmetry in variability rather than asymmetry in magnitude.

Across all pathways combined, no hemispheric differences were observed for tract count (TC), voxel count (VC), or HMOA. However, tract-specific analyses revealed heterogeneous hemispheric asymmetries in variability (see Figure 2B and Supplementary Table 3), indicating that the left and right hemispheres exhibit different levels of variability (dispersion/spread) across diffusion indices.

The inferior longitudinal fasciculus (ILF) and frontal aslant tract (FAT) showed greater variability in the left hemisphere (ILF TC: VI = −0.33, p < 0.00001; ILF VC: VI = −0.19, p = 0.0007; FAT TC: VI = −0.58, p < 0.00001). The FAT voxel count did not survive correction (VI = −0.18, p = 0.0036). In contrast, the anterior arcuate fasciculus (AFa) and uncinate fasciculus (UF) exhibited rightward variability (AFa TC: VI = 0.28, p = 0.00025; AFa VC: VI = 0.24, p = 0.00021; UF TC: VI = 0.30, p = 0.00003; UF VC: VI = 0.32, p = 0.00002). All other pathways (AFl, AFp, IFOF) showed no hemispheric asymmetry in variability across all indices (p>0.0021).

### Phenotypes of structural language lateralisation

**Figure 3.**
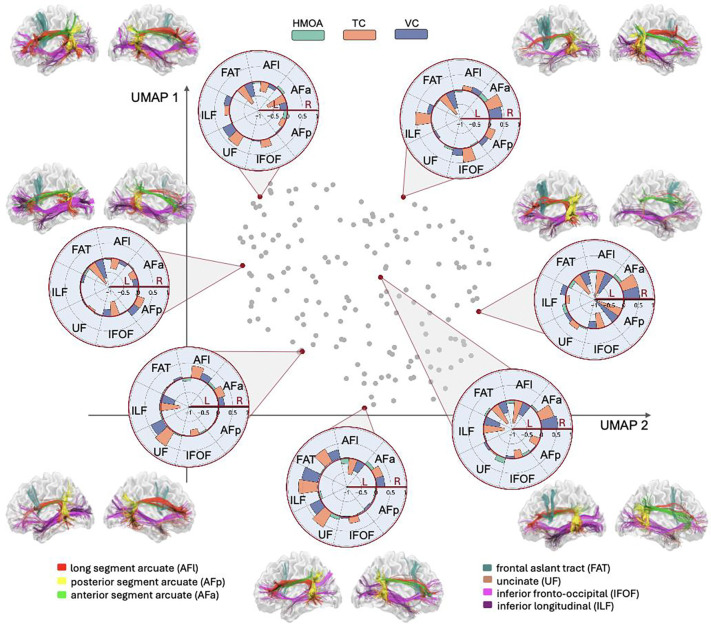
Low dimensional embedding of white matter lateralisation indices. Each point represents one participant (n = 164). The two-dimensional UMAP visualises relationships among lateralisation indices (LIs) across seven language-recruited tracts. Red points highlight exemplar participants individual tractograms and showing characteristic lateralisation patterns across hindrance-modulated orientational anisotropy (HMOA), track count (TC), and voxel count (VC): negative values (inner circle) indicate leftward (L) asymmetry, positive values (outer circle) indicate rightward (R) asymmetry. The diffuse, unclustered distribution indicates that white matter lateralisation varies along a continuous spectrum rather than forming discrete subgroups.

One aspect that both the LI and VI do not capture is whether the observed variability of a given diffusion metric reflects a single continuous distribution or multiple underlying subgroups. To test this, we applied Uniform Manifold Approximation and Projection (UMAP)^25^ to LI values across the seven language-recruited pathways. The analysis demonstrated a diffuse, unclustered topology (Figure 3), indicating that structural asymmetries vary along a smooth continuum rather than forming discrete left-, right-, or bilaterally dominant subgroups.

This manifold structure suggests that hemispheric specialisation for language white matter anatomy reflects graded individual differences rather than categorical brain phenotypes. Such a continuous organisation aligns with theories proposing that language lateralisation emerges from distributed network dynamics rather than fixed anatomical dichotomies, bridging population variability with the broader architecture of the language connectome.

### Handedness and structural lateralisation

**Figure 4.**
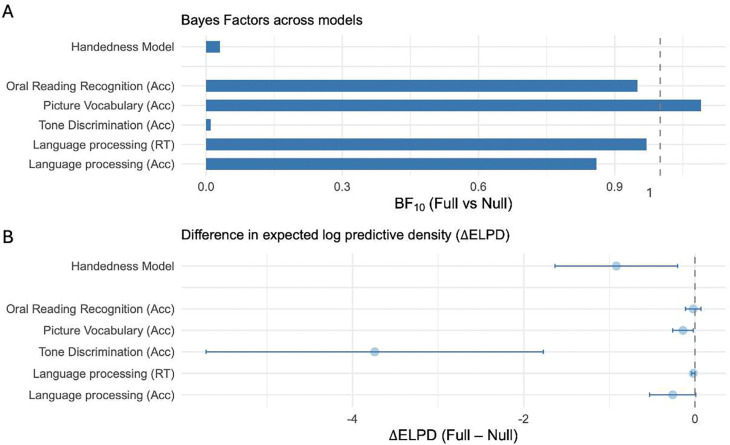
Bayesian model comparisons for handedness analysis (top row in each panel) and language tasks analysis (bottom 5 rows in each panel). The top row shows the handedness model, comparing a model with (full) and without (null) handedness as a predictor of lateralisation indices. The bottom five rows show task-level models, comparing models with (full) and without (null) tract-level lateralisation indices as predictors of behavioural performance. **A:** Bayes Factors (BF_10_) for handedness and language models. Values near 0 indicate evidence in favour of the null (handedness model and the tone discrimination model), while values near 1 indicate little or no evidence favouring either model (the rest of the behavioural tasks). **B:** Difference in expected log predictive density (ΔELPD = ELPD_full − ELPD_null), with one standard-error bars. Negative values indicate that the full model predicted unseen data slightly worse than the null model.

We tested whether tract-level lateralisation indices (LIs) were associated with handedness, as measured by the Edinburgh Handedness Inventory (EHI)^33^, employing Bayesian hierarchical mixed-effects models. Handedness did not explain additional variance in LI values: the posterior mean estimate was −0.01 (SE = 0.01, 95% CI [−0.02, 0.01]). Bayesian model comparison provided very strong evidence for the null model over the model that includes handedness (BF_10_ = 0.03), and the difference in expected log predictive density (ΔELPD) indicated negligible improvement in predictive accuracy (ΔELPD = −0.9, SE = 0.7). Together, these results provide convergent evidence that handedness is not associated with the structural lateralisation of the assessed white matter pathways in this cohort. This finding supports recent large-scale studies reporting dissociations between motor and language asymmetries^34^, suggesting that hemispheric specialisation for language arises independently of manual dominance (see top row in each panel in Figure 4).

### Structural–functional correspondence

We examined whether structural lateralisation of language-relevant white matter tracts predicts behavioural performance across four language tasks from the NIH Toolbox and Human Connectome Project: *Language Processing* (accuracy and median reaction time), *Picture Vocabulary* (audio–picture matching), *Oral Reading Recognition* (visual word decoding), and *Tone Discrimination*. A common assumption is that greater tract-level asymmetry (especially toward the left hemisphere) supports stronger language performance.

Across all models and tasks, the 95% credible intervals for all regression coefficients included zero (Supplementary Tables 4–8), indicating substantial uncertainty about the presence and direction of any association between lateralisation indices and behavioural performance. Bayesian model comparisons were consistent with this conclusion: Bayes factors were generally close to 1 (range = 0.86–1.09), suggesting that the data were largely uninformative with respect to differences between the full and null models (intercept only). The only exception was the Tone Discrimination Test, where the Bayes factor was 0.01, providing very strong evidence in favour of the null model.

Differences in expected log predictive density (ΔELPD) between full and null models were close to zero across tasks (−0.26 to −0.02), indicating minimal improvement in predictive accuracy, except for the Tone Discrimination Test (ΔELPD = −3.74), where the negative value indicated poorer predictive performance of the full model.

These results indicate that structural lateralisation alone did not meaningfully predict performance on any of the language-related behavioural measures (see bottom five rows in each panel of Figure 4).

Taken together, these findings reveal a multilayered organisation of language-recruited white matter. At the population level, major association tracts exhibit clear but tract-specific hemispheric lateralisation, reflecting central tendencies across individuals. At the same time, there is substantial interhemispheric variability, with some tracts showing greater dispersion of white matter metrics in one hemisphere than in the other across the sample. When tracts are considered jointly, they indicate that individual differences in structural lateralisation form a continuous spectrum rather than discrete left- or right-hemispheric types, as illustrated by the UMAP manifold. Finally, structural indices of lateralisation were not associated with behavioural performance, indicating that language lateralisation cannot be reduced to tract-level morphology alone.

## Discussion

This study presents the most comprehensive investigation to date of hemispheric asymmetry and interindividual variability in the white matter architecture supporting language. Our analyses converge on a consistent conclusion: structural lateralisation of language pathways forms a continuum across individuals rather than segregating into discrete subtypes. The UMAP embedding revealed a smooth gradient without distinct clusters^25^, and Bayesian statistics confirmed the significant existence or absence of tract-specific asymmetries. These findings reframe the language connectome not as a system divided into left- and right-dominant groups, but as a dynamic, continuous spectrum of hemisphere specialisation.

This continuous perspective contrasts with many functional imaging frameworks that classify participants into “typical” and “atypical” language-dominant groups^35,36^. Such categorical labels can be helpful for some clinical or group-level comparisons, but they commonly rely on arbitrary thresholds and are highly sensitive to sample composition. For example, a recent large-scale cohort (n = 995) identified 82 individuals with right-hemisphere language dominance, most of whom were right-handed^35^ — a pattern not observed in earlier healthy-cohort studies^36,37^. These discrepancies illustrate that the apparent prevalence of “atypical” profiles fluctuates with cohort size and demographics, supporting the view that functional lateralisation is better characterised as a continuum^38^.

Structural subtyping of the language connectome has been less explored than functional phenotyping. An early influential study^39^ proposed three apparent subgroups driven by asymmetry in the long segment of the arcuate fasciculus. However, those phenotypes were derived using analytical approaches that impose discrete categories on what may in fact be graded biological variation. By contrast, our findings reveal the full spectrum of interindividual structural diversity. The resulting diffuse, cluster-free topology indicates that language lateralisation is not organised into natural biological subtypes, and that discretising structural language lateralisation risks imposing artefactual categories and misrepresenting the underlying biology.

Even without discrete structural phenotypes, our analysis reveals substantial hemisphere-specific differences in dispersion that lateralisation indices alone do not capture. Rather than being defined solely by mean asymmetry, hemispheric organisation also differs in how tightly or flexibly each hemisphere is structured across individuals. For instance, the anterior segment of the arcuate fasciculus (AFa) shows group-level rightward lateralisation while simultaneously exhibiting greater dispersion within the right hemisphere. In other words, although the population bias is rightward, individuals vary widely in the magnitude of AFa characteristics on the right, whereas left-hemisphere values are relatively constrained. By contrast, the posterior and long segments of the arcuate fasciculus show comparable dispersion across hemispheres. This pattern — stable mean asymmetry together with hemisphere-specific dispersion — suggests an anatomical pattern in which conserved structural biases coexist with variable individual specialisation^40^, possibly via differential functional recruitment or asymmetric plasticity.

From an evolutionary perspective, hemisphere-specific variability may reflect distinct constraints on the organisation of language pathways. High variability within one hemisphere could indicate greater developmental or functional flexibility, supporting individual-level specialisation within an overall asymmetric architecture, whereas low variability may signal configurations shaped by strong stabilising selection^41,42^. The long segment of the arcuate fasciculus illustrates this principle: its low and balanced interhemispheric variability suggests a highly conserved architecture, consistent with evidence for substantial heritability^43^. Comparative preliminary data further support this view, with chimpanzee homologue tracts showing greater variability compared to humans^44^.

Conceptually, our analyses indicate that language pathways differ not only in the direction of their asymmetry, but in the strength and consistency of that asymmetry across the population. We demonstrated very strong evidence for leftward lateralisation in the long arcuate (AFl), inferior longitudinal fasciculus (ILF), and frontal aslant tract (FAT); very strong evidence for rightward lateralisation in the anterior arcuate (AFa) and uncinate fasciculus (UF); and mixed or bilateral patterns in posterior arcuate (AFp) and inferior fronto-occipital (IFOF) pathways depending on the diffusion metric. Importantly, some tracts (e.g. AFl) exhibit highly robust population biases, whereas others (e.g., AFp) occupy a more ambiguous position along the lateralisation continuum. Simulations manipulating sample size further indicate that tracts with comparatively weaker evidence would require substantially larger cohorts to achieve statistical stability, offering a parsimonious explanation for inconsistencies in the existing literature for certain tracts (see Supplementary Table 10 and Figure B from Supplementary Analysis 1).

The seven white matter tracts analysed are repeatedly implicated in language processing (see Supplementary Table 10). Our exploratory analyses relating structural lateralisation to available language measures from the NIH toolbox, however, yielded inconsistent associations. The lack of consistent evidence for structure–function relationships likely reflects the generality and limited sensitivity of the HCP cognitive battery for higher-order language functions, consistent with previous reports of modest anatomical–functional correlations^10,45^. Prior studies have shown dissociations between anatomical lateralisation and functional language dominance. For example, Vernooij et al. (2007) reported consistent leftward structural lateralisation of the arcuate fasciculus independent of individual functional language lateralisation. More recent tractography-fMRI work likewise failed to identify robust relationships between arcuate lateralisation and functional language dominance^45^. At the cortical level, grey matter asymmetries in classical language areas show only weak correlations with functional lateralisation^46^. Importantly, there is no one tract one function association^19^ within and across the hemispheres. As such, other functions can load on the same structure in the opposite hemisphere, leading to apparent structural symmetry despite functional asymmetry^47,48^. These convergent findings support a model in which structural lateralisation provides a scaffold, while functional specialisation emerges from distributed network dynamics that are not reducible to local structural asymmetries^31,35,48–50^.

Handedness has long been proposed as a possible moderator of structural asymmetries^51–53^. In our sample, left- or mixed-handers comprised 13.4% of participants, but including handedness did not substantially improve the explained variance in structural asymmetries. This aligns with large-scale studies reporting weak or absent associations between language lateralisation and motor asymmetries^38,54^ and with genetic evidence that handedness and language lateralisation are largely ontogenetically independent^34,55–57^. Emerging work suggests that interhemispheric connectivity (e.g., corpus callosum microstructure) may play a mediating role in shaping lateralisation patterns^45,58^.

Several limitations should be acknowledged. First, our focus was limited to a core set of well-characterised language tracts; future work should extend the analysis to additional pathways (e.g., vertical occipital fasciculus, the superior longitudinal fasciculi, commissural and projection fibres) that could contribute to language processing. Second, our cohort is drawn from young, educated adults in the HCP dataset, limiting generalisability to older, clinical, or non-majority populations^59–62^. Recent cross-cultural comparisons report substantial differences in white matter anatomy between Eastern and Western cohorts^63^, emphasising the need for broader, more diverse sampling. Finally, language is a multimodal^64–66^ and plastic system — research on sign language, Braille reading, and gestural communication shows how modality and experience reshape language networks^67–68^. An expanded definition of the language connectome that includes developmental, cultural, and multimodal diversity will be necessary to capture the full space of neurolinguistic variability.

In conclusion, the white matter scaffold of language is best understood as a continuous, multidimensional landscape of hemispheric specialisation, in which conserved population-level biases coexist with tract- and hemisphere-specific variability. This perspective moves the field beyond binary taxonomies and mean-based descriptions that obscure the full range of interindividual lateralisation, toward models that explicitly capture graded, individualised specialisation and the developmental, genetic, and experiential forces shaping variability across the population.

## Methods

### Neuroimaging Data

The study included 7T diffusion-weighted images of 172 healthy young adult participants from the Human Connectome Project; https://www.humanconnectome.org/). Data are openly available and provided by the Human Connectome Project, WU-Minn Consortium (Principal Investigators: David Van Essen and Kamil Ugurbil; 1U54MH091657), funded by the 16 NIH Institutes and Centers that support the NIH Blueprint for Neuroscience Research, and by the McDonnell Center for Systems Neuroscience at Washington University. The participants' ages averaged 29.3 ± 3.3 years, with 105 women included. The majority of the cohort was right-handed (87%), according to the Edinburgh Handedness Inventory (EHI), with 9.9% ambidextrous and 3.5% left-handed. The acquisition details can be accessed from the HCP website (https://www.humanconnectome.org/hcp-protocols-ya-7t-imaging)^69,70^. In eight participants, we could not reconstruct some pathways (details below) and excluded them from further statistical analyses, resulting in a total of 164 participants included in the statistical analysis.

### Data Preprocessing and Processing

The data underwent preprocessing using the default HCP pipeline (v3.19.0)^71,72^. To address susceptibility-induced off-resonance field distortions, pairs of images with diffusion gradients applied in opposite directions were used to estimate and correct for distortions across the entire diffusion-weighted dataset using FSL’s TOPUP^73,74^. Motion and geometric distortions were then corrected using EDDY^74–76^. The pipeline is available at https://github.com/Washington-University/Pipelines and primarily uses tools from FSL and FreeSurfer. Volumes with a b-value of 1000 s mm−2 were subsequently discarded.

Whole-brain deterministic tractography was performed on the native diffusion-weighted imaging (DWI) space using StarTrack software (https://nbl-research.github.io/). Spherical deconvolutions were applied using a damped Richardson-Lucy algorithm^16,17,78^, utilising a fixed fibre response with a shape factor of α = 1.5 × 10–3 mm2 s−1 and a geometric damping parameter. The algorithm was iterated 200 times. For tractography, an absolute threshold of three times the spherical fibre orientation distribution (FOD) of a grey matter isotropic voxel and a relative threshold of 8% of the maximum amplitude of the FOD were employed^79^. Tractography was performed using a modified Euler algorithm with an angle threshold of 35°, a step size of 0.5 mm, and a minimum streamline length of 15 mm^79^.

Several steps were followed to register the structural connectome data to the standard MNI 2 mm space. First, whole-brain streamline tractography was converted into streamline density volumes, where voxel intensities represented the number of streamlines crossing each voxel. A study-specific template of streamline density volumes was then generated using the Greedy symmetric diffeomorphic normalisation (GreedySyN) pipeline provided with ANTs^80^. This template was co-registered to a standard 2 mm MNI152 template using the FLIRT tool in FSL, resulting in a streamline density template in MNI152 space. Individual streamline density volumes were then registered to the streamline density template in the MNI152 space, and the same transformation was applied to the individual whole-brain streamline tractography using the trackmath tool distributed with the Tract Querier software package^70,81^ and ANTs GreedySyn. This step yielded a whole-brain streamline tractography in the standard MNI152 space. The preprocessed individual whole-brain connectomes are available in Radboud Data Repository at 2 mm resolution in MNI152 space (https://doi.org/10.34973/e0fm-pr30).

### White matter dissections and quantitative indices

A manual dissection approach was employed in this study. The anatomical Regions of Interest (ROIs) are aligned with previous atlases^22,82–84^. We focused on seven association tracts that have consistently been implicated in language processing across lesion studies, intraoperative mapping, and tractography research. This targeted selection balances anatomical specificity, reproducibility in high-resolution tractography, and theoretical relevance to core linguistic functions. While not exhaustive, these tracts provide a well-defined scaffold for investigating asymmetry and variability within the language connectome^19,85^. The language network, therefore, included the frontal aslant tract (FAT)^86,87^, the inferior fronto-occipital fasciculus (IFOF)^88,89^, the inferior longitudinal fasciculus (ILF)^90^, the uncinate fasciculus (UF)^83^, and the three segments of the arcuate fasciculus (AFl: long segment; AFa: anterior segment; AFp: posterior segment)^7^.

A trained master’s student (CB) performed the manual dissections of the seven bilateral tracts following structured training with a designated dataset. The training also included completing an online dissection tutorial for the three segments of the arcuate fasciculus (see tutorial: https://www.youtube.com/watch?v=PB4Cfc4EQDw). Tractography reconstructions were conducted using TrackVis (www.trackvis.org). Where necessary, experienced neuroanatomists (MTdS, SJF) reviewed and corrected discrepancies by manually refining ROI placement to ensure anatomical accuracy. In addition, the semi-automated MegaTrack software^91^ was used to perform an additional dissection cleaning step, tailored to the seven pathways (EGC, AB), to manually remove spurious streamlines from the combined tractograms.

For visualisation purposes, tractograms for each of the seven bilateral tracts and each participant were converted into voxel-wise visitation maps, where voxel intensities represented the number of streamlines passing through each voxel. These maps were binarised to reflect tract presence (1) or absence (0) per voxel. To increase spatial coherence, binarised maps were smoothed using a 1 mm FWHM Gaussian kernel and then averaged across participants.

Following individual pathway reconstruction, quantitative measures of white matter pathway properties were extracted for each tract, including hindrance-modulated orientational anisotropy (HMOA)^17^, tract count (TC), and voxel count (VC). HMOA quantifies the strength of water diffusion along a given fibre orientation within a voxel, making it a tract-specific rather than a voxel-specific measure^17^. HMOA exhibits high sensitivity to alterations in fibre diffusivity (e.g., myelination status or axonal loss) and variations in microstructural organisation (e.g., differences in axonal diameter and dispersion)^17^. Tract count represents the total number of streamlines assigned to a specific tract. Voxel count indicates the space occupied by a tract by counting the voxels intersected by a streamline. Similarly to the previous study^84^, we calculated the percentage of successful reconstruction for each tract in both hemispheres as follows:

$$Percentage\;of\;success=\frac{N\;\mathrm{of}\;\mathrm{successful}\;\mathrm{reconstructions}\;\mathrm{per}\;\mathrm{tract}}{N\;\mathrm{particiapnts}}\;*\;100$$

### Lateralisation index (LI)

To ensure methodological consistency with prior studies on white matter asymmetry^8^ and to facilitate more robust cross-study comparisons, we adopted a standardised formula. This approach maintains comparability across studies and ensures that LI values remain within the fixed range between −1 and 1, where negative values indicate left lateralisation, positive values indicate right lateralisation, and values close to zero reflect a bilateral distribution. The LI was calculated as follows: $LI=\frac{{\mathrm{value}}_{\mathrm{right}}\;-\;{\mathrm{value}}_{\mathrm{left}}}{{\mathrm{value}}_{\mathrm{right}}\;+\;{\mathrm{value}}_{\mathrm{left}}}$

To assess the lateralisation of white matter tracts statistically, we employed a Bayesian modelling framework. We evaluated whether the mean LI differed from zero (i.e., bilaterality) by comparing two models: an intercept-only model (LI ~ 1), estimating the group-level mean LI, and a null model without an intercept (LI ~ 0), assuming a mean of zero. This comparison is functionally equivalent to a one-sample t-test and was repeated across 21 variables (7 tracts × 3 metrics).

Models were estimated using the brms package in R (via Stan)^92–94^ with default settings: four Markov Chain Monte Carlo (MCMC) chains, each running 2000 iterations (including 1000 warm-up), using the No-U-Turn Sampler (NUTS). Convergence and mixing were evaluated by inspecting trace plots and verifying that all R-hat values were close to 1, indicating stable and well-mixed chains (see folder “Data_analysis/results/Bayesian/Lateralisation/mixing”, https://doi.org/10.34973/e0fm-pr30).

A widely accepted weakly informative Cauchy prior (0, 0.707)^95^ was specified for the intercept, constrained to the interval [−1, 1], to reflect the expected range of the LI. The Cauchy distribution was chosen for its heavy tails, which provide robustness to outliers while allowing for a range of effect sizes from small to large. The scale parameter of 0.707 represents a standard “medium effect” prior that balances informativeness with flexibility. This choice aligns with the defaults used in tools such as JASP^96^ and the BayesFactor R package^97^, enhancing comparability across analyses.

To assess the sensitivity of our findings to prior assumptions (i.e., robustness), we re-estimated each model using a range of Cauchy prior widths (0.01 to 1), confirming that Bayes factors and posterior estimates remained stable. Bayes factors were calculated using the bayes_factor() function in brms, comparing the alternative (intercept) model to the null model. Statistical analysis was performed in RStudio (R version 4.2.2^98^, and the script is publicly available (https://doi.org/10.34973/e0fm-pr30). For reference, results from a frequentist one-sample t-test are provided in Supplementary Analysis 1.

### Variability Index (VI)

The Variability Index (VI) represents the *relative interindividual dispersion* (i.e., hemispheric asymmetry in variability) in white matter tract properties between hemispheres. Specifically, it quantifies which hemisphere shows more variability across individuals for a given metric (e.g., tract count, volume, HMOA), rather than which hemisphere has a higher *mean* value (as the LI does). It is computed using median absolute deviation (MAD), a robust statistical measure of spread that is less sensitive to outliers and skewed distributions than standard deviation.

For example, if the left long segment of the arcuate fasciculus has a higher mean HMOA than the right, the LI would reflect leftward lateralisation. In contrast, the VI answers a different question: which hemisphere exhibits *greater variability* across individuals in the sample? If the left FAT shows widely varying tract counts (with some individuals having very small or large values), the VI would identify the left FAT as more variable than the right. The distinction between the two indices is crucial, as the LI focuses on central tendency (mean differences) in the sample, while the VI focuses on dispersion (variability). These are orthogonal but complementary measures.

To robustly operationalise variability, we employed the median absolute deviation (MAD), which is more resistant to outliers and non-normal distributions than the standard deviation. We computed MAD separately for the left and right hemispheres across individuals for each tract. The VI was then calculated as follows: $VI=\frac{{\mathrm{MAD}}_{\mathrm{right}}\;-\;{\mathrm{MAD}}_{\mathrm{left}}}{{\mathrm{MAD}}_{\mathrm{right}}\;+\;{\mathrm{MAD}}_{\mathrm{left}}}$, where MAD is the median of absolute deviations from the dataset’s median, representing intrahemispheric variability (i.e., variability within a hemisphere across individuals). A negative VI indicates greater left-hemisphere variability than the right hemisphere, a positive value reflects greater right-hemisphere variability, and zero denotes equal variability between the two hemispheres.

We employed the Monte Carlo permutation method to assess the statistical significance of the variability^24^. Since the data contains paired within-participant measures (left and right hemispheres per participant), the permutation procedure used in this study preserves the within-participant dependency structure by randomly swapping the left and right values within each participant with a 50% probability. For each permutation (total n = 100.000), the variability index was recalculated, generating a null hypothesis distribution of VI values and positing that there are no systematic hemispheric variability differences. Next, we computed the absolute proportion of permuted differences equal to or more extreme than the actual observed value, yielding a p-value.

Additionally, we computed hemisphere-averaged (across all tracts) VI indices. We applied the same permutation-based approach to examine variance differences at the hemispheric level per index (i.e., HMOA, TC, VC). Bonferroni's alpha threshold was corrected to 0.0021 (0.05 / [7 tracts × 3 measures] + 3 global left vs. right comparisons).

### Handedness and Lateralisation

To evaluate whether LI values were associated with handedness, as measured by the Edinburgh Handedness Inventory (EHI)^33^, we fit Bayesian hierarchical mixed models using the brms package. Seven tracts and three metrics were included as categorical predictors, while handedness (rescaled from its original −100 to 100 range to −1 to 1 for numerical stability) was included as a continuous predictor. Random intercepts were specified for participants to account for repeated measures across tracts and metrics. Two models were estimated: a null model excluding handedness, and a full model including handedness as a main effect. Both models were fit with the default Gaussian likelihood and priors, using four Markov Chain Monte Carlo (MCMC) chains, each with 2000 iterations (including 1000 warm-up iterations). Convergence and mixing were assessed via trace plots and R-hat statistics, confirming stable and well-mixed chains. Evidence for the inclusion of handedness was quantified using Bayes factors, while predictive performance was evaluated using leave-one-out cross-validation (LOO), allowing us to assess whether adding handedness improved the model’s ability to predict unseen data.

### Behavioural and Cognitive Language Measures

We assessed whether structural lateralisation of language-recruited white matter tracts was related to performance on language tasks. Behavioural data were drawn from the NIH Toolbox included in the Human Connectome Project (HCP) dataset (https://www.nihtoolbox.org/)^99^. From the language domain, we analysed the Picture Vocabulary Test (auditory word–image matching) and the Oral Reading Recognition Test (visual word decoding). As a control for auditory perception, we included the Tone Discrimination Test. In addition, we examined the Language Processing Task^100^, which involved listening to short stories followed by comprehension questions, providing both accuracy and median reaction time measures.

For each behavioural outcome, we fit Bayesian regression models in brms with default settings (4 chains × 2000 iterations). The full model included all tract lateralisation indices (LIs) as predictors, while the null model contained only an intercept. Coefficients were assigned weakly informative truncated Cauchy (0, 0.707) priors bounded between −1 and 1 to respect the theoretical bounds of LI values. Evidence for associations was evaluated using posterior 95% credible intervals, Bayes factors (full vs. null), and leave-one-out cross-validation (LOO) for model comparison (full vs. null).

### Lateralisation Phenotypes

To explore potential clustering patterns among participants based on white matter lateralisation, we applied a non-linear dimensionality reduction technique, Uniform Manifold Approximation and Projection (UMAP)^25^ in Python using the umap-learn library (version 0.5.9.post2). The analysis was performed on the full set of LI values across all tracts and metrics with UMAP default settings (n_neighbors = 15, min_dist = 0.1, Euclidean distance metric). The resulting two-dimensional embedding was plotted to qualitatively assess whether participants formed distinct clusters.

### Systematic Review

We reviewed existing white matter atlases encompassing language-relevant pathways and extracted key methodological features for comparison, including study cohort, imaging modality, tractography algorithm, manual versus automated dissection, and the specific tracts reported. This allowed us to identify which of the seven language-relevant tracts analysed in the present study were represented or missing across previous atlases. The review covered 27 atlases published since 2002 (see Supplementary Table 9).

## Supplementary Material

This is a list of supplementary files associated with this preprint. Click to download.

• DataRepository.docx

• Supplementarymaterial.pdf

## Figures and Tables

**Table 1.** Results of the Bayesian analysis of lateralisation.

Metric	Tract	Est. Mean	Est. Error	95%CI	Bayes Factor
HMOA	AFl	−0.077	0.004	[−0.08, −0.07]	9.6 × 10^36
TC	AFl	−0.226	0.031	[−0.29, −0.16]	4.69 × 10^8
VC	AFl	−0.147	0.020	[−0.19, −0.11]	1.04 × 10^9
HMOA	AFa	0.100	0.006	[0.09, 0.11]	4.58 × 10^30
TC	AFa	0.244	0.037	[0.17, 0.31]	2.69 × 10^7
VC	AFa	0.322	0.027	[0.27, 0.37]	2.52 × 10^21
HMOA	AFp	−0.013	0.005	[−0.02, 0]	0.44
TC	AFp	−0.107	0.033	[−0.17, −0.04]	11.48
VC	AFp	−0.081	0.023	[−0.13, −0.04]	20.18
HMOA	IFOF	−0.028	0.003	[−0.03, −0.02]	3.59 × 10^10
TC	IFOF	−0.003	0.028	[−0.06, 0.05]	0.05
VC	IFOF	−0.024	0.016	[−0.06, 0.01]	0.09
HMOA	UF	0.073	0.007	[0.06, 0.09]	3.42 × 10^14
TC	UF	0.323	0.029	[0.27, 0.38]	4.64 × 10^18
VC	UF	0.326	0.023	[0.28, 0.37]	1.62 × 10^27
HMOA	ILF	−0.033	0.005	[−0.04, −0.02]	2.43 × 10^6
TC	ILF	−0.271	0.028	[−0.33, −0.22]	2.83 × 10^14
VC	ILF	−0.167	0.020	[−0.21, −0.13]	1.21 × 10^11
HMOA	FAT	−0.023	0.004	[−0.03, −0.02]	9.35×10^4
TC	FAT	−0.550	0.026	[−0.6, −0.5]	2.97 × 10^44
VC	FAT	−0.341	0.024	[−0.39, −0.29]	4.4 × 10^26

AFl: long segment of arcuate fasciculus, AFa: anterior segment of arcuate fasciculus, AFp: posterior segment of arcuate fasciculus, IFOF: inferior fronto-occipital fasciculus, UF: uncinate fasciculus, ILF: inferior longitudinal fasciculus, FAT: frontal aslant tract, HMOA: hindrance modulated orientational anisotropy, TC: track count, VC: voxel count, 95%CI: 95% credible interval, Est. Mean: estimated mean, Est. Error, estimated error.

## Data Availability

The preprocessed individual whole-brain connectomes for 172 Human Connectome Project Participants, manually dissected language-recruited white matter pathways for each participant, tract-specific quantitative metrics, behavioural measures, analysis scripts, and statistical analysis outputs are available from Radboud Data Repository (https://doi.org/10.34973/e0fm-pr30). Tractography tutorials 3 segments: https://youtu.be/ypUSTD-5p1Q, advanced tractography: https://www.youtube.com/live/l0gBaXIsyWQ?feature=share.
